# Characteristics and quality of systematic reviews led by Peruvian authors: A scoping review

**DOI:** 10.1016/j.heliyon.2024.e36887

**Published:** 2024-08-24

**Authors:** Ana Brañez-Condorena, David R. Soriano-Moreno, Jhonatan R. Mejia, Lesly Chavez-Rimache, Daniel Fernandez-Guzman, Raisa N. Martinez-Rivera, Naysha Becerra-Chauca, Carolina J. Delgado-Flores, Alvaro Taype-Rondan

**Affiliations:** a EviSalud - Evidencias en Salud, Lima, Peru; b Unidad de Investigación Clínica y Epidemiológica, Escuela de Medicina, Universidad Peruana Unión, Lima, Peru; c Carrera de Medicina Humana, Universidad Científica del Sur, Lima, Peru; d Unidad de Investigación para la Generación y Síntesis de Evidencias en Salud, Vicerrectorado de Investigación, Universidad San Ignacio de Loyola, Lima, Peru; e Sociedad Científica de Estudiantes de Medicina de la Universidad Nacional de Piura (SOCIEMUNP), Piura, Peru; f Facultad de Salud Pública y Administración, Universidad Peruana Cayetano Heredia, Lima, Peru; g Carrera de Farmacia y Bioquímica, Facultad de Ciencias de la Salud, Universidad Científica del Sur, Lima, Peru

**Keywords:** Systematic review, Peru, AMSTAR 2

## Abstract

**Background:**

Systematic reviews (SRs) worldwide suffer from methodological deficiencies, potentially biasing intervention decisions, and Peruvian SRs are no exception. Evaluating SRs led by Peruvian researchers is a crucial step to enhance quality and transparency in decision-making and to identify topics where SRs are either scarce or prioritized for research.

**Objective:**

To describe the characteristics and assess the methodological quality of SRs with Peruvian first authors.

**Methods:**

We conducted a scoping review within the Scopus database on January 5, 2023. We aimed to identify published SRs of interventions in which the first author had a Peruvian affiliation, published between 2013 and 2022. We evaluated the methodological quality of these SRs using the AMSTAR 2 tool. We assessed the factors associated with the AMSTAR 2 score using adjusted mean differences (aMD), including their 95 % confidence intervals (95 % CI).

**Results:**

We identified 95 eligible SRs, with a clear upward trend. SRs were primarily published in Q1 (43.2 %) and Q2 (23.2 %) journals, predominantly affiliated with institutions in Lima (90.5 %). Areas like infectious diseases (20.0 %) and dentistry (18.9 %) were most frequent. AMSTAR 2 assessments highlighted deficiencies, with few SRs reporting prior protocols (37.9 %), comprehensive search strategies (23.2 %), explanations for excluded studies (20.0 %), adequate descriptions of included studies (38.3 %), or funding sources (19.1 %). Notably, SRs in Q4 journals (aMD: −19.7, 95 % CI: −33.8 to −5.5) and those on surgical interventions (aMD: −22.6, 95 % CI: −34.7 to −10.4) had lower AMSTAR 2 scores.

**Conclusions:**

Although Peruvian-led SRs are increasingly being published, critical deficiencies are common, especially in reporting protocols, search strategies, study descriptions, and funding sources. Addressing these gaps is pivotal for enhancing the credibility and utility of these SRs in informing decision-making.

## 1 Introduction

Systematic reviews (SRs) represent a cornerstone in the scientific literature by comprehensively analyzing available evidence on a specific research question in a systematic and replicable approach [1]. This study design has emerged as a vital resource for decision-making in healthcare, underpinning evidence-based health policies and clinical practice [2]. The quality of SRs is critical to ensure confidence in their results and their applicability to the informed decision-making process [3].

Previous studies have assessed the methodological quality of SRs to guide their correct elaboration [[4], [5], [6], [7], [8]] utilizing A Measurement Tool to Assess Systematic Reviews (AMSTAR) tool, first published in 2007 [9] and with an updated version in 2017 (AMSTAR 2) [10]. These tools have been employed in prior studies to identify methodological deficiencies in several healthcare areas, including psychiatry [5,11], endocrinology [12], neurology [4], rheumatology [7], surgery [8,13,14], and telemedicine [6].

Nonetheless, there remains a scarcity of research specifically targeting the assessment of SRs authored by researchers from diverse geographical regions or countries. Studying this gap is valuable for researchers, funders, and readers alike; since it can aid in pinpointing specific methodological areas that could benefit from improvement, uncovering factors influencing the methodological quality of SRs, and recognizing topics of interest for the country where SRs are conspicuously scarce or are listed as research priorities.

This approach has been applied in evaluating SRs within the Eastern Mediterranean region [15] and Korea [16], highlighting the importance of extending such investigations to different global contexts to comprehensively assess SRs quality and characteristics across diverse authorship backgrounds. In the Peruvian context, quality assessment of intervention SRs is needed along with an exploration of topics where SRs are scarce or that constitutes the national research priorities, such as non-communicable diseases, anemia, malnutrition, and mental health [17].

Thus, this study was conducted to describe the characteristics and assess the quality of SRs published by Peruvian first authors in Scopus. The results may lead to recommendations to research groups and relevant stakeholders in Peru to improve the overall quality of Peruvian SRs.

## 2 Material and methods

We conducted a scoping review following the Preferred Reporting Items for Systematic Reviews and Meta-Analyses for Scoping Reviews (PRISMA-ScR) guidelines [18] to ensure adequate reporting of the study.

### 2.1 Eligibility criteria

We included studies that fulfilled the following criteria: 1) SRs with or without meta-analysis, 2) The first author had any affiliation from Peru, 3) Addressed an intervention question with a control group and a human population, and 4) Were published in a scientific journal between 2013 and 2022 (last 10 years). Focusing on the first author indicates that the primary contribution was from Peruvian researchers. The time frame from 2013 to 2022 was selected to capture recent trends, reflecting the current state of evidence and practices. We included only intervention SRs to achieve a homogeneous evaluation of methodological characteristics.

We excluded previous versions of SRs and other types of reviews (e.g. case report reviews, umbrella reviews, and scoping reviews). No restrictions on language were applied.

### 2.2 Search strategy and study selection

We performed a search in the Scopus database from 2013 to January 5, 2023; using the following search strategy: AFFILCOUNTRY(PERU) AND (TITLE-ABS-KEY("systematic review") OR TITLE-ABS-KEY("meta-analysis") OR TITLE-ABS-KEY("metaanalysis") OR TITLE-ABS-KEY("metanalysis")).

Two pairs of authors (DRSM, RNMR, JRM, and NBC) independently selected the SRs that met the eligibility criteria for inclusion. In case of discrepancies, a third researcher (ATR) was consulted.

### 2.3 Data collection

Four pairs of authors (DRSM, RNMR, JRM, NBC, ABC, CDF, LCR, and DFG) independently extracted the following information from included SRs using a standardized Microsoft Excel sheet: 1) Characteristics of the SR (year of publication, covered areas, type of intervention, sources of funding), 2) Methodological characteristics (search, primary studies selection, data collection, use of meta-analyses, report of a minimally important difference (MID)), 3) Use of *Grading of Recommendations, Assessment, Development, and Evaluations* (GRADE) methodology through the report of Summary of Findings (SoF) table [19], 4) Publication characteristics (English-language journal, best annual quartile [Q1 - Q4] according to Scimago [20]), 5) Number of all authors and those with Peruvian affiliation, and 6) Characteristics of affiliations (Peruvian departments, Peruvian institutions, multi-affiliation). Disagreements were resolved by discussion or, if necessary, another researcher was consulted (ATR).

### 2.4 Quality assessment

Four pairs of authors (DRSM, RNMR, JRM, NBC, ABC, CDF, LCR, and DFG) independently assessed the quality of SRs using AMSTAR 2 tool. Disagreements were resolved by discussion or, if necessary, another researcher was consulted (ATR).

The AMSTAR 2, extensively employed for assessing the methodological quality of SRs, evaluates different aspects of a SR through 16 items [10]. The fulfillment of all 16 items was assessed if the SR performed a meta-analysis. For SRs that did not perform meta-analyses, the fulfillment of AMSTAR 2 was performed with the evaluation of 13 items (since items 11, 12, and 15 require meta-analyses). Furthermore, assessments vary for each SR depending on the feasibility of evaluating certain items. For instance, item 15 (publication bias) was evaluated only in SRs with a meta-analysis involving at least 10 primary studies.

Each SR was classified according to the AMSTAR 2 categories [10] in the results of the review, as high confidence (no or one non-critical weakness), moderate confidence (more than one non-critical weakness), low confidence (one critical flaw with or without non-critical weaknesses), and critically low confidence (more than one critical flaw with or without non-critical weaknesses).

Additionally, an overall AMSTAR 2 score was calculated for each SR, by dividing the number of completed items by the total number of assessed items. An item was considered completed if the response was ‘yes,' while it was deemed incomplete if the responses were “no”, “partial yes” or “not applicable”.

### 2.5 Statistical analyses

Statistical analyses were performed using Stata v.17 software. Categorical variables were expressed as frequencies and percentages, and numerical variables were expressed as measures of central tendency and dispersion. The percentage of fulfillment was obtained for each AMSTAR 2 item.

In addition, an exploratory analysis was performed to assess the factors associated with overall AMSTAR 2 scores. For this analysis, we calculated crude and adjusted mean differences (MD and aMD) and their 95 % confidence intervals (95 % CI) using crude and adjusted linear regressions. We conducted a sensitivity analysis to evaluate the factors associated with critically low confidence in AMSTAR 2 [10]. This analysis involved reporting crude and adjusted prevalence ratios (PR and aPR) with their 95 % CI using Poisson regressions with robust variance. The adjusted model included all variables that had a p-value <0.20 in the crude model. A p-value <0.05 was considered statistically significant.

## 3 Results

**Fig. 1:**
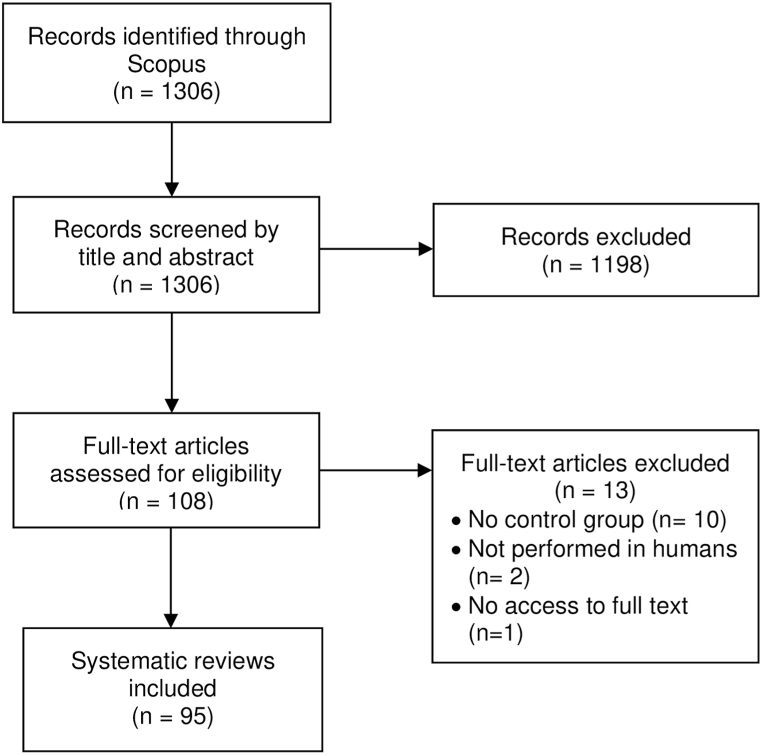
Flowchart of systematic review selection.

**Fig. 2:**
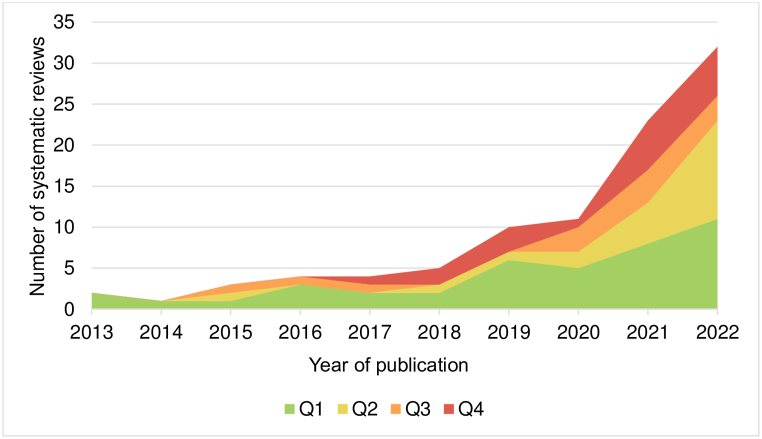
Number of systematic reviews with Peruvian first authors in Scopus (2013–2022), per Scimago quartiles. Q1: top 25 %, Q2: top 26%–50 %, Q3: top 51%–75 %, Q4: top 76%–100 %.

A total of 1306 records were identified in the Scopus database, of which 95 SRs published between 2013 and 2022 met the inclusion criteria (Fig. 1). A rising trend in SR publications by Peruvian authors was observed. These studies were published predominantly in Q1 (43.2 %) and Q2 (23.2 %) journals (Fig. 2).

### 3.1 Studies characteristics

The majority of all Peruvian authors involved in SR publications were affiliated with institutions located in the region of Lima (90.5 %), followed by La Libertad (21.1 %) and Lambayeque (14.7 %). Concerning all author-affiliated institutions, the University San Ignacio de Loyola led with 29.5 % of contributions, followed by Universidad Peruana Cayetano Heredia (23.2 %), Universidad de San Martín de Porres (22.1 %), and Universidad Científica del Sur (18.9 %). Regarding author team characteristics, the median number of authors per study was 6, with a median of 4 authors with some affiliation in Peru.

Regarding characteristics of first authors and their affiliations, most first authors had a single affiliation (55.8 %), while a significant percentage had two (31.6 %) or three affiliations (12.6 %). Geographically, Lima again led with 82.1 % of the first authors' primary affiliations, followed by Lambayeque (11.6 %) and La Libertad (6.3 %). In 28.4 % of the SRs, at least one author had both Peruvian and foreign affiliation (international multi-affiliations) (Table 1).

**Table 1:** Filiation characteristics of systematic reviews with Peruvian first authors in Scopus (2013–2022).

Characteristics	n (%)
Region of Peru involved according to the affiliation of the authors^a^	
Lima	86 (90.5)
La Libertad	20 (21.1)
Lambayeque	14 (14.7)
Piura	3 (3.2)
Cajamarca	2 (2.1)
Ancash	1 (1.1)
Tumbes	1 (1.1)
Institution most frequently involved according to authors' affiliations^a^	
Universidad San Ignacio de Loyola	28 (29.5)
Universidad Peruana Cayetano Heredia	22 (23.2)
Universidad San Martín de Porres	21 (22.1)
Universidad Científica del Sur	18 (18.9)
Universidad Nacional Mayor de San Marcos	15 (15.8)
Universidad Peruana de Ciencias Aplicadas	15 (15.8)
Number of Peruvian institutions in affiliations^b^,^c^	3 (1–4)
Number of authors^b^,^c^	6 (4–8)
Number of authors with any affiliation from Peru^b^,^c^	4 (3–6)
Some author with a Peruvian and a foreign affiliation	27 (28.4)
Number of affiliations of the first author	
1	53 (55.8)
2	30 (31.6)
3	12 (12.6)
Region of Peru corresponding to the first author's first affiliation	
Lima	78 (82.1)
Lambayeque	11 (11.6)
La Libertad	6 (6.3)

a Each systematic review could be in one or more categories.

b Median (interquartile range).

c Per systematic review.

It was observed that 79.0 % of SRs were published in English-language journals, and 53.7 % were self-funded. Concerning study selection, 57.9 % of SRs included only randomized controlled trials (RCTs). The most frequent covered areas were infectious diseases (20.0 %), dentistry (18.9 %), gynecology and obstetrics (7.4 %), and cardiology (7.4 %). Concerning the types of interventions evaluated, pharmaceuticals were the primary focus in 54.7 % of SRs. The number of studies finally included in the SRs varied considerably, with a median of 10 studies (IQR: 6 to 17) studies. Additionally, 75.8 % of SRs conducted meta-analyses, but only 39.0 % of SRs reported a Summary of Findings (SoF) table, and 1.1 % of SRs established a MID for at least one variable (Table 2).

**Table 2:** Characteristics of systematic reviews with Peruvian first authors in Scopus (2013–2022).

Characteristics	n (%)
Published in an English-language scientific journal	75 (79.0)
Funding	
Funded	23 (24.2)
Self-funded	51 (53.7)
Not specified	21 (22.1)
Type of studies finally included	
RCT only	55 (57.9)
Non-RCT only	11 (11.6)
RCT and non-RCT	28 (29.5)
Did not include any studies	1 (1.1)
Areas most frequently addressed	
Infectology	19 (20.0)
Dentistry	18 (18.9)
Gynecology and Obstetrics	7 (7.4)
Cardiology	7 (7.4)
Type of intervention evaluated	
Drugs	52 (54.7)
Public health interventions	14 (14.7)
Surgical interventions	11 (11.6)
Devices	8 (8.4)
Others	10 (10.5)
Number of search engines or databases^a^	5 (4–7)
Number of studies finally included^a^	10 (6–17)
Performs meta-analysis	72 (75.8)
Reports SoF table	37 (39.0)
Establishes a MID for at least one variable	1 (1.1)
AMSTAR 2 assessment	
High confidence	4 (4.2)
Moderate confidence	3 (3.2)
Low confidence	11 (11.6)
Critically low confidence	77 (81.1)

MID: minimal important difference, RCT: randomized clinical trial, Non-RCT: Non-randomized clinical trial, SoF: summary of findings.

a Median (interquartile range).

### 3.2 AMSTAR 2 assessment

The majority of the SRs (81.1 %) had critically low confidence according to AMSTAR 2 (Table 2). The evaluation of the AMSTAR 2 criteria indicated that only 38.3 % of the SRs adequately described the included studies (item 8). Furthermore, only 37.9 % of the SRs explicitly stated that the methods had been established before conducting the review and provided justifications for deviations (item 2). Aspects such as the use of an exhaustive literature search strategy (23.2 %, item 4), the presentation of a list of excluded studies with justifications (20.0 %, item 7), and the report of the funding sources of the included studies (19.1 %, item 10) showed the lowest percentage of compliance. However, 97.9 % of the authors reported any conflict of interest (item 16), 88.4 % performed duplicate study selection (item 5), and 85.9 % satisfactorily explained any heterogeneity observed in the results (item 14) (Table 3).

**Table 3:** Assessment of the included systematic reviews using the AMSTAR 2 items (2013–2022).

AMSTAR 2 items	n/N (%)
1. Did the research questions and inclusion criteria for the review include the components of PICO?	75/95 (78.9)
2. Did the report of the review contain an explicit statement that the review methods were established prior to the conduct of the review and did the report justify any significant deviations from the protocol?	36/95 (37.9)
3. Did the review authors explain their selection of the study designs for inclusion in the review?	55/95 (57.9)
4. Did the review authors use a comprehensive literature search strategy?	22/95 (23.2)
5. Did the review authors perform study selection in duplicate?	84/95 (88.4)
6. Did the review authors perform data extraction in duplicate?	75/94 (79.8)
7. Did the review authors provide a list of excluded studies and justify the exclusions?	19/95 (20.0)
8. Did the review authors describe the included studies in adequate detail?	36/94 (38.3)
9. Did the review authors use a satisfactory technique for assessing the risk of bias in individual studies that were included in the review?	78/94 (83.0)
10. Did the review authors report on the sources of funding for the studies included in the review?	18/94 (19.1)
11. If meta-analysis was performed did the review authors use appropriate methods for statistical combination of results?	52/72 (72.2)
12. If meta-analysis was performed, did the review authors assess the potential impact of risk of bias in individual studies on the results of the meta-analysis or other evidence synthesis?	33/72 (45.8)
13. Did the review authors account for risk of bias in individual studies when interpreting/discussing the results of the review?	42/71 (59.2)
14. Did the review authors provide a satisfactory explanation for, and discussion of, any heterogeneity observed in the results of the review?	55/64 (85.9)
15. If they performed quantitative synthesis did the review authors carry out an adequate investigation of publication bias (small study bias) and discuss its likely impact on the results of the review?	20/25 (80.0)
16. Did the review authors report any potential sources of conflict of interest, including any funding they received for conducting the review?	93/95 (97.9)

n: number of systematic reviews that fulfilled the assessed item, N: total number of systematic reviews in which it was possible to assess the item.

Among the factors associated with the AMSTAR 2 score, the adjusted analyses revealed that SRs published in Q4 journals (aMD: −19.7; 95 % CI: −33.8 to −5.5) and those evaluating surgical interventions (aMD: −22.6; 95 % CI: −34.7 to −10.4) were associated with lower scores compared to SRs published in Q1 journals and those evaluating pharmacological interventions, respectively (Table 4). These findings were consistent in the sensitivity analysis (Table 5), which showed that the prevalence of critically low confidence was higher in the SRs published in Q4 journals (aPR: 1.61; 95 % CI: 1.15 to 2.24) and those evaluating surgical interventions (aPR: 1.23; 95 % CI: 1.03 to 1.48) compared to SRs published in Q1 journals and those evaluating pharmacological interventions, respectively. Additionally, the SRs with 6–7 authors (aPR: 0.71; 95 % CI: 0.53 to 0.96) and those with 1–5 authors (aPR: 0.73; 95 % CI: 0.56 to 0.94) had a lower prevalence of critically low confidence compared to SRs with 8–15 authors.

**Table 4:** Factors associated with the AMSTAR 2 score.

Characteristics	n	Score in AMSTAR 2 Mean ± standard deviation	MD (95 % CI)	aMD (95 % CI)^a^
Year of publication				
2013 to 2018	19	66.8 ± 24.1	9.6 (−2.3 to 21.6)	5.4 (−7.5 to 18.2)
2019 to 2020	21	49.1 ± 23.3	−8.0 (−19.3 to 3.2)	−5.5 (−19.1 to 8.1)
2021 to 2022	55	57.1 ± 18.7	Ref	Ref
Scimago quartile				
Q1	41	66.1 ± 20.5	Ref	Ref
Q2	22	54.9 ± 22.2	−11.2 (−22.6 to 0.2)	−8.4 (−18.8 to 2.1)
Q3	13	51.2 ± 24.3	**−14.9 (-29.5 to -0.3)**	−11.6 (−27.5 to 4.2)
Q4	19	45.2 ± 12.0	**−21.0 (-29.4 to -12.6)**	**−19.7 (-33.8 to -5.9)**
Department of Peru corresponding to the first author's first affiliation				
Lima	78	58.7 ± 22.3	Ref	Ref
Not Lima	17	51.1 ± 16.7	−7.6 (−16.9 to 1.7)	−1.9 (−15.1 to 11.2)
Number of authors				
1 to 5	44	52.3 ± 25.2	**−10.5 (-19.8 to -1.3)**	−0.9 (−12.0 to 10.3)
6 to 7	25	60.2 ± 19.6	−2.6 (−12.1 to 6.8)	3.4 (−7.4 to 14.1)
8 to 15	26	62.9 ± 13.8	Ref	Ref
Funding				
Funded	23	58.7 ± 24.0	Ref	Ref
Self-funded	51	60.1 ± 19.3	1.33 (−9.9 to 12.6)	6.26 (−8.2 to 20.7)
Not specified	21	49.0 ± 22.5	−9.75 (−23.6 to 4.1)	−0.64 (−18.2 to 16.9)
Area most frequently addressed				
Infectology	19	56.5 ± 19.2	Ref	
Dentistry	18	54.0 ± 12.5	−2.41 (−12.8 to 8.0)	
Others	58	58.6 ± 24.3	2.12 (−8.6 to 12.9)	
Type of intervention evaluated				
Drugs	52	62.8 ± 19.5	Ref	Ref
Public health interventions	14	58.9 ± 24.3	−3.95 (−17.8 to 9.9)	−3.21 (−15.6 to 9.2)
Surgical interventions	11	38.6 ± 15.8	**−24.26 (-35.0 to -13.5)**	**−22.55 (-34.7 to -10.4)**
Devices	8	53.1 ± 19.6	−9.70 (−24.0 to 4.6)	−7.53 (−22.6 to 7.5)
Others	10	50.2 ± 23.9	−12.58 (−28.2 to 3.0)	−8.97 (−25.3 to 7.3)

95 % CI: 95 % confidence interval, MD: crude mean difference, aMD: adjusted mean difference.

Q1: top 25 %, Q2: top 26%–50 %, Q3: top 51%–75 %, Q4: top 76%–100 %.

a The adjusted model contains all the variables shown in this column.

**Table 5:** Factors associated with critically low confidence according to AMSTAR 2.

Characteristics	n	Confidence according to AMSTAR 2		PR (95 % CI)	aPR (95 % CI)^a^
		High, moderate or low	Critically low		
		n (%)	n (%)		
Year of publication					
2013 to 2018	19	7 (36.8)	12 (63.2)	0.72 (0.50–1.04)	0.75 (0.52–1.09)
2019 to 2020	21	4 (19.0)	17 (81.0)	0.93 (0.74–1.17)	0.91 (0.71–1.18)
2021 to 2022	55	7 (12.7)	48 (87.3)	Ref	Ref
Scimago quartile					
Q1	41	12 (29.3)	29 (70.7)	Ref	Ref
Q2	22	4 (18.2)	18 (81.8)	1.16 (0.87–1.53)	1.20 (0.89–1.61)
Q3	13	2 (15.4)	11 (84.6)	1.20 (0.88–1.62)	1.25 (0.90–1.75)
Q4	19	0 (0.0)	19 (100.0)	**1.41 (1.16 to 1.72)**	**1.61 (1.15 to 2.24)**
Department of Peru corresponding to the first author's first affiliation					
Lima	78	17 (21.8)	61 (78.2)	Ref	Ref
Not Lima	17	1 (5.9)	16 (94.1)	**1.20 (1.02 to 1.42)**	1.00 (0.78–1.28)
Number of authors					
1 to 5	44	10 (22.7)	34 (77.3)	0.84 (0.69–1.02)	**0.73 (0.56 to 0.94)**
6 to 7	25	6 (24.0)	19 (76.0)	0.82 (0.64–1.06)	**0.71 (0.53 to 0.96)**
8 to 15	26	2 (7.7)	24 (92.3)	Ref	Ref
Funding					
Funded	23	6 (26.1)	17 (73.9)	Ref	Ref
Self-funded	51	11 (21.6)	40 (78.4)	1.06 (0.80–1.41)	0.87 (0.60–1.25)
Not specified	21	1 (4.8)	20 (95.2)	1.29 (0.99–1.68)	0.92 (0.61–1.40)
Area most frequently addressed					
Infectology	19	3 (15.8)	16 (84.2)	Ref	
Dentistry	18	2 (11.1)	16 (88.9)	1.06 (0.82–1.36)	
Others	58	13 (22.4)	45 (77.6)	0.92 (0.72–1.17)	
Type of intervention evaluated					
Drugs	52	9 (17.3)	43 (82.7)	Ref	Ref
Public health interventions	14	5 (35.7)	9 (64.3)	0.78 (0.51–1.17)	0.81 (0.54–1.22)
Surgical interventions	11	0 (0.0)	11 (100.0)	**1.21 (1.07 to 1.37)**	**1.23 (1.03 to 1.48)**
Devices	8	2 (25.0)	6 (75.0)	0.91 (0.60–1.38)	1.02 (0.66–1.58)
Others	10	2 (20.0)	8 (80.0)	0.97 (0.69–1.35)	1.04 (0.71–1.52)

95 % CI: 95 % confidence interval, PR: crude prevalence ratio, aPR: adjusted prevalence ratio.

Q1: top 25 %, Q2: top 26%–50 %, Q3: top 51%–75 %, Q4: top 76%–100 %.

a The adjusted model contains all the variables shown in this column.

## 4 Discussion

SRs of interventions led by Peruvian authors were published mainly in Q1 and Q2 journals, predominantly affiliated with institutions in Lima. The most frequent areas were infectious diseases and dentistry. AMSTAR 2 evidenced that few SRs reported previous protocols, adequate descriptions of the included studies, comprehensive search strategies, explanations of excluded studies, or funding sources. SRs published in Q4 journals and those related to surgical interventions had lower AMSTAR 2 scores.

We found that, according to the AMSTAR 2 tool, 81.1 % of the SRs received a critically low confidence while 11.6 % had a low confidence. This is similar to a study that assessed the methodological quality of SRs on non-communicable diseases in the Eastern Mediterranean region and found that 83.2 % of the SRs received a low confidence rating [15], and a study focused on SRs of nursing interventions in Korea which found that 90.9 % exhibited low or moderate confidence [16]. Additionally, 88.9 % of SRs from Europe, Asia, North and South America related to chronic prostatitis or chronic pelvic pain were rated as critically low quality [21]. Most previous studies show a lack of adherence to protocol registration (item 2), statement of selection of the study design (item 3), exclusion reasons (item 7), funding of individual studies (item 10), and discussion of the impact of risk of bias (item 13) [15,16,21]. Altogether, these findings indicate that concerns about methodological quality are not limited to a specific region but span an international spectrum.

Notably, even SRs published in high-impact journals are not exempt from this issue [22]. Additionally, the accelerated production of evidence in emergency situations, such as the COVID-19 pandemic, has led to 87.7 % of SRs on this topic receiving a low or critically low confidence rating [23].

Regarding each item of the AMSTAR 2 checklist, there were some concerns about deviations from the protocol, comprehensive search strategies, reasons for the exclusion of studies, adequate descriptions of included studies, and funding sources.

Concerning deviations from the protocol, only one-third of the authors registered their SRs or published their protocols, this proportion is similar to previous studies [[24], [25], [26], [27], [28]]. One reason could be that the authors may not have been aware of the recording of their protocols. Furthermore, some journals do not require the registration of protocols, which may be more common among those with lower impact factors [13,28].

Regarding a comprehensive search strategy, only 23.2 % of the SRs were fulfilled, similar to a previous study that focused on erectile dysfunction treatments [29]. The absence of full search strategies could be because AMSTAR 2 considers keyword presence sufficient, though full strategies are recommended for reproducibility [30,31].

Only 20 % of the SR authors reported an explanation for study exclusions, following the trend of previous studies [11,29,32,33]. This item is important to detect any study selection bias; however, the question could be interpreted as requiring the author to justify all the records or only those read in full text, which could be the main reason for under-reporting [30].

A detailed description of included studies was fulfilled in only 38.3 % of SRs. Other studies focusing on treatments for major depression [11] and erectile dysfunction [29] in adults reported rates of 22 % and 43.1 %, respectively. It is a challenge to comply with this item because there are no thresholds to decide if the information reported in the characteristics of the studies is detailed, ultimately this depends on the user's aim or the objective of the SR [30].

Only 19.1 % of the SRs reported the source of funding for the included studies, consistent with other research showing percentages between 6.9 % and 25 % [11,12,14,29,33]. This is a minor criterion in the AMSTAR 2 tool, which may contribute to its underreporting. Nonetheless, recognizing potential biases introduced by study funders is crucial, as it can affect the integrity of the presented results [10].

Additionally, we evaluated the association of different factors and the overall AMSTAR 2 score, considering that lower scores indicate lower adherence to methodological standards [10]. A lower AMSTAR 2 score was associated with journals having a lower impact factor (Q4), and studies related to surgery, compared to SRs published in Q1 journals and those evaluating pharmacological interventions, respectively. Higher-impact journals are more likely to enforce rigorous compliance with the PRISMA and AMSTAR 2 guidelines, resulting in better reporting of methodological quality, as reported in other studies [13,34,35].

Only 39 % of SRs included a SoF table and one SR has established a MID, despite PRISMA 2020 specifies that SRs should report the certainty of the evidence [36], and GRADE recommends performing a partial contextualization based on the MID [37]. This poor reporting of the SRs is likely because the GRADE and PRISMA 2020 guidelines are relatively recent. Further analyses should evaluate whether adherence to these guidelines improves in SRs authored by Peruvian researchers.

The highest production of SRs was observed in institutions in Lima. Ninety percent of SRs have an author with an affiliation in Lima, 82 % of them being the first author. In addition, the six institutions with the most published SRs were in Lima. This predominance of Lima has been previously reported in studies on HIV/AIDS [38] and dentistry [39].

Caution is required when reporting authors' affiliations, as we have found a higher number of multiple affiliations. This probably results from the lack of standardized criteria for when to use an institution as affiliation, and because several Peruvian institutions offer publication bonuses [40]. Notably, 31.6 % of the SRs included had first authors with dual affiliation, and 28.4 % even had international multi-affiliation (affiliations in more than one country). These scenarios complicate the analysis of potential collaborative networks because SRs with different affiliations might reflect multi-affiliations rather than actual collaborations.

According to our results, Peruvian researchers should strive for better adherence to 10.13039/501100009129SR protocols by addressing deviations, providing comprehensive search strategies, clearly stating reasons for study exclusion, adequately describing included studies, and disclosing funding sources. Additionally, they should consider a partial contextualization approach when assessing the certainty of the evidence, reporting MIDs, and designing SoF tables in line with the GRADE approach. Adhering to these AMSTAR 2 items and the GRADE methodology would enhance the reliability of SR results, thereby providing more robust information for decision-making processes in clinical practice guidelines, health technology assessments, and everyday clinical practice. On the other hand, it is necessary to maintain the positive trends in reporting of conflict of interest, conducting duplicate study selection, and reporting heterogeneity. Further research is needed to develop focused strategies that guide Peruvian researchers in adhering to SR quality assessment tools and GRADE methodology. Additionally, while the most frequently addressed areas by Peruvian SR authors were infectology, dentistry, gynecology, obstetrics, and cardiology, there is a notable gap in SRs related to mental health. Addressing this gap is essential for aligning with national research priorities.

### 4.1 Limitations and strengths

We should consider the following limitations: 1) We used only the Scopus database for the SR search, and we did not include other regional databases such as LILACS and SciELO which lack the capability to filter by affiliation and limits their suitability for our specific research scope. Scopus is quite well recognized as it presents a wide range of articles, offers advanced tools for searching metadata by affiliation, and provides English abstracts for SRs originally published in Spanish. 2) Although AMSTAR 2 proposes a categorization based on the fulfillment of critical items, we believe that some of these critical items as the justification of excluded studies (item 7) may not always be important for methodological quality classification. Evaluating the factors associated with critically low confidence showed that most SRs were categorized as having low or critically low confidence, leading to statistically unstable results. This instability arose because some cells in the data table showed a prevalence of high, moderate, or low confidence at 0 %. Therefore, we decided to use the sum of the number of fulfilled AMSTAR 2 items as the main exploratory analysis. While acknowledging that not all items carry the same weight, we believe that their sum provides a comprehensive assessment of the overall quality of SRs. 3) We focused on SR with a first author with Peruvian affiliation as this suggests a primary contribution was from Peruvian researchers. However, the role of the last author, often considered a senior leader, should also be acknowledged. The designation of the last author as a senior leader is not always clearly defined and can vary widely. In some instances, the last author might be an external collaborator or someone who contributed less to the research. Due to these variations and the challenges in accurately determining the role of the last author, we did not consider this position. In addition, we acknowledge the contribution of foreigners affiliated with Peruvian institutions; however, there is not enough information that suggests the research leadership came from Peru, as foreign leadership may also be a factor, and 4) On the other hand, we cannot be sure that the included SRs are not involved in scientific misconduct (e.g. purchase of authorship, authorship gifts, etc.).

However, this scoping review has the following strengths: 1) the processes of study selection, data extraction and evaluation by AMSTAR 2 were developed in duplicate, 2) the AMSTAR 2 was used, which is a widely used instrument for the evaluation of the methodological quality of the SR, and 3) Relevant information was collected from the SRs, such as the use of the GRADE methodology through the SoF table report and the MID report, among others.

## 5 Conclusion

In conclusion, although Peruvian-led SRs are being published increasingly, critical deficiencies are common, especially in reporting protocols, search strategies, study descriptions, and funding sources. Addressing these gaps is pivotal for enhancing the credibility and utility of these SRs in informing decision-making.

## Funding

This study was self-funded.

## Ethics statement

Review and/or approval by an ethics committee was not needed for this study because it does not involve primary data collection from human participants.

## Data availability statement

Data included in article/supp. Material/referenced in article.

## CRediT authorship contribution statement

**Ana Brañez-Condorena:** Writing – review & editing, Writing – original draft, Methodology, Investigation, Data curation. **David R. Soriano-Moreno:** Writing – review & editing, Writing – original draft, Methodology, Data curation, Conceptualization. **Jhonatan R. Mejia:** Writing – review & editing, Writing – original draft, Methodology, Data curation, Conceptualization. **Lesly Chavez-Rimache:** Writing – review & editing, Writing – original draft, Methodology, Data curation. **Daniel Fernandez-Guzman:** Writing – review & editing, Writing – original draft, Methodology, Data curation. **Raisa N. Martinez-Rivera:** Writing – review & editing, Writing – original draft, Methodology, Data curation, Conceptualization. **Naysha Becerra-Chauca:** Writing – review & editing, Writing – original draft, Methodology, Data curation, Conceptualization. **Carolina J. Delgado-Flores:** Writing – review & editing, Writing – original draft, Methodology, Data curation. **Alvaro Taype-Rondan:** Writing – review & editing, Writing – original draft, Supervision, Methodology, Investigation, Formal analysis, Conceptualization.

## Declaration of competing interest

The authors declare the following financial interests/personal relationships which may be considered as potential competing interests: Ana Brañez-Condorena, Lesly Chavez-Rimache, Naysha Becerra-Chauca, Carolina J. Delgado-Flores, and Alvaro Taype-Rondan conducted nine systematic reviews that were included in the study. However, these authors did not assess their own systematic reviews. The study selection, data extraction, and AMSTAR 2 assessments were performed by the other authors. The authors declare no additional potential conflicts of interest related to this study.
